# Supplementary material for: Impact of spectacle use on academic performance among Vietnamese adolescents with reduced visual acuity and myopia: A school-based study
Source: PLoS One. 2025 May 12;20(5):e0322534. doi: 10.1371/journal.pone.0322534 (PMC12068628; doi:10.1371/journal.pone.0322534)
Supplement: Attachment — Submitted filename: PONE-D-24-39987_reviewer comments.pdf [file pone.0322534.s002.pdf]

PLOS ONE

The impact of eyeglasses on academic performance among Vietnamese adolescents with low vision and myopia: a school-based study

--Manuscript Draft--

**Manuscript Number:**	PONE-D-24-39987
**Article Type:**	Research Article
**Full Title:**	The impact of eyeglasses on academic performance among Vietnamese adolescents with low vision and myopia: a school-based study
**Short Title:**	Wearing glasses and academic performance among adolescents in Vietnam with low vision
**Corresponding Author:**	Keiko Nakamura<br>Tokyo Medical and Dental University: Tokyo Ika Shika Daigaku<br>Tokyo, JAPAN
**Keywords:**	academic performance; Adolescents; glass-wearing; low vision; myopia
**Abstract:**	Objectives: The World Heal[illegible]rganization’s VISION 2020: The Right to Sight initiative prioritized reducing uncorrected refractive errors and avoidable childhood blindness. Prompt correction of these vision issues improves both visual function and quality of life. This study assessed the effects of wearing glasses on the academic performance of school students with low vision and myopia.<br>Methods: This cross-sectional study included 647 students from five secondary schools in Hue City, Vietnam. Students underwent comprehensive eye examinations including visual acuity, autorefractometry, and axial length measurements, alongside a structured questionnaire. Academic performance was measured using grade point average (GPA), math, and literature scores. Generalized linear models were utilized to examine the association between wearing glasses and students’ academic performance.<br>Results: The mean spherical equivalent was -0.92 ± 1.62 diopters, and the mean axial length was 23.32 ± 1.07 mm. The prevalence of low vision (uncorrected visual acuity ≤ 7/10) and myopia (spherical equivalent ≤ -0.5 diopters) was 23.2% and 48.7%, respectively. Almost two-thirds of myopic students did not wear glasses. Wearing glasses was found to be significantly associated with increased grade point averages (β = 0.462; 95% confidence interval = 0.108–0.816), math scores (β = 0.517; 95% confidence interval = 0.015–1.020), and literature scores (β = 0.438; 95% confidence interval = 0.074–0.802) in students with low vision, and increased literature scores (β = 0.277; 95% confidence interval = 0.046–0.509) in students with myopia.<br>Conclusions: Refractive errors, especially myopia, are significant public health issues among Vietnamese secondary school students. Although those students are expected to wear proper glasses to correct refractive errors, a large proportion of them do not which is expected to lead to their vision problems. This study underscores the benefits of wearing glasses among students with low vision and myopia for improving academic performance.
**Order of Authors:**	Hoang Thuy Linh Nguyen, MD, MPH, PhD
	Xuan Minh Tri Tran
	Keiko Nakamura, MD, PhD
	Kaoruko Seino, PhD
	Yuri Tashiro
	Ayano Miyashita
	Tae Igarashi-Yokoi
	Van Thang Vo
	Kyoko Ohno-Matsui
**Additional Information:**	

Powered by Editorial Manager® and ProduXion Manager® from Aries Systems Corporation

Question	Response
**Financial Disclosure**<br><br>Enter a financial disclosure statement that describes the sources of funding for the work included in this submission. Review the submission guidelines for detailed requirements. View published research articles from *PLOS ONE* for specific examples.<br><br>This statement is required for submission and **will appear in the published article** if the submission is accepted. Please make sure it is accurate.<br><br>**Funded studies**<br>Enter a statement with the following details:<br>• Initials of the authors who received each award<br>• Grant numbers awarded to each author<br>• The full name of each funder<br>• URL of each funder website<br>• Did the sponsors or funders play any role in the study design, data collection and analysis, decision to publish, or preparation of the manuscript?<br><br>Did you receive funding for this work?	Yes
Please add funding details.<br>as follow-up to "**Financial Disclosure**<br><br>Enter a financial disclosure statement that describes the sources of funding for the work included in this submission. Review the submission guidelines for detailed requirements. View published research articles from *PLOS ONE* for specific examples.<br><br>This statement is required for submission and **will appear in the published article** if the submission is accepted. Please make sure it is accurate.	This work is partly supported by Japanese Society for Promotion of Science Grant (17H02164). Japanese Society for Promotion of Science has no involvement in the study design; collection, analysis and interpretation of data; and writing the manuscript.

Powered by Editorial Manager® and ProduXion Manager® from Aries Systems Corporation

**Funded studies**<br>Enter a statement with the following details:<br>• Initials of the authors who received each award<br>• Grant numbers awarded to each author<br>• The full name of each funder<br>• URL of each funder website<br>• Did the sponsors or funders play any role in the study design, data collection and analysis, decision to publish, or preparation of the manuscript?<br><br>Did you receive funding for this work?"	
Please select the country of your main research funder (please select carefully as in some cases this is used in fee calculation).<br>as follow-up to "**Financial Disclosure**<br><br>Enter a financial disclosure statement that describes the sources of funding for the work included in this submission. Review the submission guidelines for detailed requirements. View published research articles from *PLOS ONE* for specific examples.<br><br>This statement is required for submission and **will appear in the published article** if the submission is accepted. Please make sure it is accurate.<br><br>**Funded studies**<br>Enter a statement with the following details:<br>• Initials of the authors who received each award<br>• Grant numbers awarded to each author<br>• The full name of each funder<br>• URL of each funder website<br>• Did the sponsors or funders play any role in the study design, data collection and analysis, decision to publish, or preparation of the manuscript?<br><br>Did you receive funding for this work?"	JAPAN - JP
**Competing Interests**	Authors declare that the research was conducted in the absence of any commercial or

Use the instructions below to enter a competing interest statement for this submission. On behalf of all authors, disclose any competing interests that could be perceived to bias this work—acknowledging all financial support and any other relevant financial or non-financial competing interests.<br><br>This statement is required for submission and **will appear in the published article** if the submission is accepted. Please make sure it is accurate and that any funding sources listed in your Funding Information later in the submission form are also declared in your Financial Disclosure statement.<br><br>View published research articles from *PLOS ONE* for specific examples.<br><br>**NO authors have competing interests**<br><br>Enter: *The authors have declared that no competing interests exist.*<br><br>**Authors with competing interests**<br><br>Enter competing interest details beginning with this statement:<br><br>*I have read the journal's policy and the authors of this manuscript have the following competing interests: [insert competing interests here]*<br><br>* typeset	financial relationships that could be construed as a potential conflict of interest.
**Ethics Statement**<br><br>Enter an ethics statement for this submission. This statement is required if the study involved:<br><br>• Human participants<br>• Human specimens or tissue<br>• Vertebrate animals or cephalopods<br>• Vertebrate embryos or tissues	The protocol for this study was approved by the institutional review boards of the medical schools of Tokyo Medical and Dental University, Japan (M2017-176), and Hue University of Medicine and Pharmacy, Vietnam (January 6th, 2018). Permission to recruit secondary school students was obtained from the Department of Education and Training in Thua Thien Hue Province, Vietnam. The investigative team prescheduled a meeting with the principals of the randomly selected schools and received consent before the onset of the study. All of the principals agreed to participate. All of the enrolled subjects agreed to participate after the purpose of the study was explained, and written informed consent was obtained from the parents/guardians and study subjects before their participation.

• Field research<br><br>Write "N/A" if the submission does not require an ethics statement.<br><br>General guidance is provided below. Consult the submission guidelines for detailed instructions. **Make sure that all information entered here is included in the Methods section of the manuscript.**	

**Format for specific study types**<br><br>**Human Subject Research (involving human participants and/or tissue)**<br>• Give the name of the institutional review board or ethics committee that approved the study<br>• Include the approval number and/or a statement indicating approval of this research<br>• Indicate the form of consent obtained (written/oral) or the reason that consent was not obtained (e.g. the data were analyzed anonymously)<br><br>**Animal Research (involving vertebrate animals, embryos or tissues)**<br>• Provide the name of the Institutional Animal Care and Use Committee (IACUC) or other relevant ethics board that reviewed the study protocol, and indicate whether they approved this research or granted a formal waiver of ethical approval<br>• Include an approval number if one was obtained<br>• If the study involved *non-human primates*, add *additional details* about animal welfare and steps taken to ameliorate suffering<br>• If anesthesia, euthanasia, or any kind of animal sacrifice is part of the study, include briefly which substances and/or methods were applied<br><br>**Field Research**<br><br>Include the following details if this study involves the collection of plant, animal, or other materials from a natural setting:<br>• Field permit number<br>• Name of the institution or relevant body that granted permission	
**Data Availability**<br><br>Authors are required to make all data underlying the findings described fully available, without restriction, and from the time of publication. PLOS allows rare exceptions to address legal and ethical concerns. See the PLOS Data Policy and FAQ for detailed information.	No - some restrictions will apply

Powered by Editorial Manager® and ProduXion Manager® from Aries Systems Corporation

A Data Availability Statement describing where the data can be found is required at submission. Your answers to this question constitute the Data Availability Statement and **will be published in the article**, if accepted.<br><br>**Important:** Stating 'data available on request from the author' is not sufficient. If your data are only available upon request, select 'No' for the first question and explain your exceptional situation in the text box.<br><br>Do the authors confirm that all data underlying the findings described in their manuscript are fully available without restriction?	
**Describe where the data may be found in full sentences. If you are copying our sample text, replace any instances of XXX with the appropriate details.**<br><br>• If the data are **held or will be held in a public repository**, include URLs, accession numbers or DOIs. If this information will only be available after acceptance, indicate this by ticking the box below. For example: *All XXX files are available from the XXX database (accession number(s) XXX, XXX.).*<br>• If the data are all contained **within the manuscript and/or Supporting Information files**, enter the following: *All relevant data are within the manuscript and its Supporting Information files.*<br>• If neither of these applies but you are able to provide **details of access elsewhere**, with or without limitations, please do so. For example:<br><br>*Data cannot be shared publicly because of [XXX]. Data are available from the XXX Institutional Data Access / Ethics Committee (contact via XXX) for researchers who meet the criteria for access to confidential data.*<br><br>*The data underlying the results presented in the study are available from (include the name of the third party*	The generated dataset is available upon request from the corresponding author at the indicated contact address.

and contact information or URL).<br>• This text is appropriate if the data are owned by a third party and authors do not have permission to share the data.<br><br>* typeset	
Additional data availability information:	

Manuscript

Click here to access/download;Manuscript;Manuscript_12Sep2024_FINAL.d

The impact of eyeglasses on academic performance among Vietnamese adolescents with low vision and myopia: a school-based study

Short title: Wearing glasses and academic performance among adolescents in Vietnam with low vision

Authors: Hoang Thuy Linh Nguyen[1], Xuan Minh Tri Tran[1,2], Keiko Nakamura[2*], Kaoruko Seino[2,3], Yuri Tashiro[2], Ayano Miyashita[2], Tae Igarashi-Yokoi[4], Van Thang Vo[1], Kyoko Ohno-Matsui[4]

Affiliations:

[1]Faculty of Public Health, Hue University of Medicine and Pharmacy, Hue University, Vietnam.

[2]Department of Global Health Entrepreneurship, Division of Public Health, Graduate School of Tokyo Medical and Dental University, Japan.

[3]National Institute of Public Health, Japan.

[4]Department of Ophthalmology and Visual Science, Tokyo Medical and Dental University, Japan.

***Corresponding author:**

Phone: +81 3 5803 4048

Fax: +81 3 5803 4034

Email: nakamura.ith@tmd.ac.jp

Abstract

Objectives: The World Health Organization's VISION 2020: The Right to Sight initiative prioritized reducing uncorrected refractive errors and avoidable childhood blindness. Prompt correction of these vision issues improves both visual function and quality of life. This study assessed the effects of wearing glasses on the academic performance of school students with low vision and myopia.

Methods: This cross-sectional study included 647 students from five secondary schools in Hue City, Vietnam. Students underwent comprehensive eye examinations including visual acuity, autorefractometry, and axial length measurements, alongside a structured questionnaire. Academic performance was measured using grade point average (GPA), math, and literature scores. Generalized linear models were utilized to examine the association between wearing glasses and students' academic performance.

Results: The mean spherical equivalent was -0.92 ± 1.62 diopters, and the mean axial length was 23.32 ± 1.07 mm. The prevalence of low vision (uncorrected visual acuity ≤ 7/10) and myopia (spherical equivalent ≤ -0.5 diopters) was 23.2% and 48.7%, respectively. Almost two-thirds of myopic students did not wear glasses. Wearing glasses was found to be significantly associated with increased grade point averages (β = 0.462; 95% confidence interval = 0.108–0.816), math scores (β = 0.517; 95% confidence interval = 0.015–1.020), and literature scores (β = 0.438; 95% confidence interval = 0.074–0.802) in students with low vision, and increased literature scores (β = 0.277; 95% confidence interval = 0.046–0.509) in students with myopia.

Conclusions: Refractive errors, especially myopia, are significant public health issues among Vietnamese secondary school students. Although those students are expected to wear proper glasses to correct refractive errors, a large proportion of them do not which is expected to lead to their vision problems. This study underscores the benefits of wearing glasses among students with low vision and myopia for improving academic performance.

Introduction

Myopia is one of the most common refractive errors and a global public health problem leading to visual impairment and blinding complications. Several epidemiological studies have suggested that approximately 10–20% of school-aged children in low- and middle-income countries have myopia [1]. The Refractive Error Study in Children (RESC) was conducted using the same sampling strategies, procedures, and definitions of myopia in different countries. The results showed varying prevalences of myopia across different populations, which were alarmingly higher in selected regions of Asia (36.3% among 7–9 years in Singapore and 42.4% among 13–17 years in China). According to the Vietnam National Plan for Blindness and Prevention and Eye Care, uncorrected refractive error (36.4%) is a major cause of childhood blindness in Vietnam and is estimated to affect 15–20% of Vietnamese children [2].

Although a substantial proportion of myopia cases are the result of genetic inheritance, this does not exclude strong environmental influences or play an important role in the determination of refractive errors, especially myopia [3]. Researchers have recently directed their focus to not only exploring risk factors, but also understanding the potential roles of protective parameters [4–8]. Interestingly, controversial or unclear data still exist that explain potential or modifiable risk factors in developing countries or rural areas [9].

The reduction of uncorrected refractive errors and avoidable blindness in children is a major priority of the World Health Organization's VISION 2020: The Right to Sight [10], as well as one of the priorities of the Vietnam National Plan. The timely correction of refractive errors can improve productivity and functionality, which enhances participation in activities of daily living and visual function. It is well known that refractive surgeries, along with the use of glasses and/or contact lenses, is an obvious solution for correcting refractive errors. Glasses provide a safe and inexpensive option for the treatment of refractive errors. Lower scores have been documented on a variety of motor and cognitive tests in children with uncorrected refractive errors; however, reading, and subsequently the aforementioned scores, may improve when vision problems are corrected. To date, there have been no studies evaluating the effects of wearing glasses on the activities of children who have refractive errors, specifically myopia, in Vietnam. Providing evidence of these effects may improve compliance with wearing glasses and prompt families to seek treatment. This study, therefore, aimed

to determine the effects of wearing glasses on academic performance among secondary school students with low vision and myopia.

Material and methods

Study population

This study was conducted in Hue City, in the central area of Thua Thien Hue Province in central Vietnam. Participants were selected from a baseline survey of an ongoing school-based cohort study ("Hue Healthy Adolescent Cohort Study") from 2018–2021. Hue City encompasses an area of 266 square kilometers and has a population of approximately 70,000 secondary-school students. Participants were selected using a multistage stratified cluster random sampling design. First, 5 secondary schools were randomly selected from among a total of 23 public secondary schools in Hue City. Second, depending on the size of the school, 4 or 5 sixth grade classes (11-year-old students) were randomly chosen from each school. The participants were recruited during the period from January 6, 2018, to February 5, 2018. The sample size of the baseline data used in a cross-sectional survey design study was 755 students. Of the 755 students invited to participate in the survey, the valid response rate was 85.70% (647 of 755).

Procedures

All study participants underwent a comprehensive eye examination, which included the measurement of visual acuity, autorefractometry, and assessment of axial length (AL). Visual acuity was tested both monocularly and binocularly at 5 m using a Landolt C chart under room light (approximately 100–300 lux). Autorefractometry was performed in a noncycloplegic state using an autorefractometer (autorefractor ARK-1; Nidek, Aichi, Japan). AL was measured with an ultrasound biometer A-scan (ECHOScan US-4000; Nidek, Aichi, Japan) using the contact method. Prior to measuring the AL, corneal anesthesia was performed with one drop of 0.5% proparacaine hydrochloride (Alcaine 0.5%; Alcon Laboratories, Fort Worth, TX, USA) to minimize discomfort. All examinations were performed by trained ophthalmologists.

Each student also completed a structured questionnaire, developed at the Department of Global Health Entrepreneurship of Tokyo Medical and Dental University, based on the Global School-Based Student Health

Survey (GSHS) and Child Refractive Error Risk Factor Questionnaire, in a face-to-face interview by a trained team of staff. The questionnaire was initially developed in English, translated to Vietnamese, and back-translated to English to verify its clarity and validity. The questions cover a broad range of activities of daily living, including study habits, time spent on electronic devices for pleasure such as playing games, and social media (Facebook, Instagram, YouTube) use.

Data analysis and definitions

Uncorrected visual acuity (UCVA) was categorized into normal (UCVA > 7/10 line) and low (UCVA ≤ 7/10) vision. The prevalence of low vision was calculated based on the UCVA in both eyes. The spherical equivalent (SE) was calculated as sphere + ½ cylinder. Myopia, emmetropia, and hyperopia were defined as ≤ -0.5 diopters (D) of SE, -0.5 D < SE < 0.5 D, and ≥ 0.5 D of SE, respectively. Only data from the right eye were used, as there was a high correlation in the SE between the right and left eyes ($r_{Spearman} = 0.87$, $P < 0.001$).

Total study time was defined as the sum of the time spent in extra classes and studying at school and home and was recorded as "< 6 hours/day," "6–8 hours/day," or "> 8 hours/day". The daily time spent on electronic devices for pleasure was calculated using the following formula: $[(time\ spent\ on\ weekdays) \times 5 + (time\ spent\ on\ weekends \times 2)]/7$. These results were recorded and classified as "not at all," "< 1 hour," and "> 1 hour."

In the descriptive analyses, categorical variables were summarized using proportions and presented in tables. The Chi-squared test (categorical variables) and Mann-Whitney U-test (continuous variables of non-normal distribution) were used to examine sex differences (boys and girls) among participants' characteristics. Generalized linear models were utilized for continuous variables (grade point average [GPA], math and literature scores) to examine the association between wearing glasses and academic performance among students with low vision and myopia (using a cut-off point of -0.5) after adjusting for sex, study time, and time spent on electronic devices for pleasure. Data were analyzed using SPSS software (IBM SPSS Statistics for Windows, Version 22.0. Armonk, NY: IBM Corp), with statistical significance set at $P < 0.05$. No imputation was performed for the missing data.

Informed consent and ethical considerations

The protocol for this study was approved by the institutional review boards of the medical schools of Tokyo Medical and Dental University, Japan (M2017-176), and Hue University of Medicine and Pharmacy, Vietnam (January 6th, 2018). Permission to recruit secondary school students was obtained from the Department of Education and Training in Thua Thien Hue Province, Vietnam. The investigative team prescheduled a meeting with the principals of the randomly selected schools and received consent before the onset of the study. All of the principals agreed to participate. All of the enrolled subjects agreed to participate after the purpose of the study was explained, and written informed consent was obtained from the parents/guardians and study subjects before their participation.

Results

The participants' characteristics are presented in Table 1. A total of 647 (85.70%) of the 755 secondary school students (11year-olds) invited participated in the study, among which 57.3% were girls while the majority lived with both parents (88.0%). The mean SE and AL of the right eye were -0.92 ±1.62 D and 23.32 ± 1.07 mm, respectively. Statistically significant differences were observed between the mean SE and AL according to sex in both eyes ($P < 0.05$). The prevalence of low vision in both eyes was 23.2%, with girls having a slightly higher prevalence than boys (13.3% vs. 9.9%, respectively; $P < 0.05$).

Table 1: Participant characteristics (n = 647)

		n (Mean)	% (SD)	*P*-value
Prevalence of low vision in both eyes[c] (%)				
	Boys	64	9.9	0.006[a]
	Girls	86	13.3	
	Total	150	23.2	
Prevalence of low vision in right eye (%)				
	Boys	76	11.7	0.001[a]
	Girls	104	16.1	
	Total	180	27.8	
Prevalence of low vision in left eye (%)				
	Boys	76	11.7	0.002[a]
	Girls	103	15.9	
	Total	179	27.7	
Spherical equivalent in right eye (D)				
	Boys	-0.72	1.45	0.00[b]
	Girls	-1.14	1.77	
	Total	-0.92	1.62	
Spherical equivalent i[illegible]t eye (D)				0.010[b]

Boys	-0.72	1.49	
Girls	-1.07	1.94	
Total	-0.89	1.72	
Axial length in right eye (mm)			
Boys	23.49	0.96	<0.001[b]
Girls	23.13	1.14	
Total	23.32	1.07	
Missing case	2		
Axial length in left eye (mm)			
Boys	23.59	0.97	<0.001[b]
Girls	23.28	0.94	
Total	23.44	0.97	
Missing case	2		

[a]Pearson Chi-squared
[b]Mann-Whitney U test
[c]Low vision was defined as ≤ 0.7 using the Landolt C chart
SD, standard deviation

Table 2 shows that the prevalence of wearing glasses among the study population was 20.6%. The prevalence of students who reported not wearing glasses was 25.3% and 63.2% among students with low vision in both eyes and those with myopia, respectively. Furthermore, the results presented in Table 3 revealed that among the low vision population (n = 150), 94.0% of the students had myopia, compared to 35.0% among those without low vision (n = 497).

Table 2: Characteristics of wearing glasses among the study population (n = 647)

		Wearing glasses				
		Yes	%	No	%	Total
**Low vision[c]**						
	Yes	112	74.7	38	25.3	150
	No	21	4.2	476	95.8	497
**Refractive error**						
	Myopia	116	36.8	199	63.2	315
	Emmetropia	10	3.4	283	96.6	293
	Hyperopia	7	17.9	32	82.1	39

[c]Low vision was defined as ≤ 0.7 using the Landolt C chart

Table 3: Distribution of low vision and refractive error (n=647)

Low vision[c]	n (%)	Refractor error	n (%)	% Total
Yes	150 (23.2)	Myopia	141 (94.0)	21.8
		Emmetropia	6 (4.0)	0.9
		Hyperopia	3 (2.0)	0.5
No	497(76.8)	Myopia	174 (35.0)	26.9
		Emmetropia	287 (57.7)	44.4
		Hyperopia	36 (7.2)	5.6

[c]Low vision was defined as ≤ 0.7 using the Landolt C chart

Table 4 shows that wearing glasses was significantly associated with academic performance in students with low vision and myopia, with a significant increase in GPA (β = 0.470; *P* = 0.012), math score (β = 0.534; *P* = 0.040), and Vietnamese literature score (β = 0.441; *P* = 0.020) compared to no increase in the univariate linear regression model. After adjusting for sex, study time, and time spent on electronic devices for pleasure, wearing glasses remained significantly associated with an increased GPA (β = 0.422; *P* = 0.011), math score (β=0.517; *P* = 0.044), and Vietnamese literature score (β=0.438; *P* = 0.018). Among the students with myopia, wearing glasses increased GPA (β = 0.237; *P* = 0.045) and Vietnamese literature (β = 0.319; *P* = 0.010) compared to students without myopia. This is consistent with the results of the multivariate linear regression (models 1 and 2) which indicated that students with myopia who wore glasses had higher scores in Vietnamese literature than students without myopia after adjusting for sex, study time, and time spent on electronic devices for pleasure (β = 0.277; *P* = 0.019).

Table 4: Factors associated with academic performance among adolescents by low vision and myopia.

	**GPA**		**Math score**		**Literature score**	
	β (95% CI)	***P*-value**	β (95% CI)	***P*-value**	β (95% CI)	**P**
**Low Vision (n=150)**						
Wearing glasses	0.470 (0.105–0.835)	0.012	0.534 (0.026–1.042)	0.040	0.441 (0.068–0.813)	0.020
**Model 1**						
Sex	-0.341 (-0.658 to-0.024)	0.035	-0.155 (-0.603–0.293)	0.497	-0.283 (-0.610–0.044)	0.090
Study time (ref. > 8 hours/day)						
≤ 6 hours/day	-0.375 (-1.153–0.403)	0.345	-0.392 (-1.493–0.708)	0.485	0.025 (-0.778–0.828)	0.952
6-8 hours/day	-0.314 (-0.670–0.041)	0.083	-0.450 (-0.953–0.052)	0.079	-0.192 (-0.558–0.175)	0.306
Wearing glasses	0.448 (0.092–0.805)	0.014	0.502 (-0.002–1.005)	0.051	0.430 (0.063–0.797)	0.022
**Model 2**						

Sex	-0.381 (-0.699 to -0.062)	0.019	-0.196 (-0.648–0.256)	0.395	-0.323 (-0.651–0.004)	0.053
Study time (ref. > 8 hours/day)						
≤ 6 hours/day	-0.439 (-1.225–0.348)	0.274	-0.441 (-1.557–0.675)	0.439	-0.098 (-0.906–0.710)	0.812
6-8 hours/day	-0.345 (-0.710–0.020)	0.064	-0.471 (-0.989–0.046)	0.074	-0.260 (-0.635–0.115)	0.174
Time spent electronic devices for pleasure (ref. > 1 hours/day)						
Not at all	-0.752 (-2.110–0.606)	0.278	-0,859 (-2.787–1.068)	0.382	-0.481 (-1.877–0.914)	0.499
≤ 1 hours/day	-0.984 (-2.383–0.415)	0.168	-1.053 (-3.039–0.932)	0.298	-0.873 (-2.311–0.565)	0.224
Wearing glasses	0.462 (0.108–0.816)	0.011	0.517 (0.015–1.020)	0.044	0.438 (0.074–0.802)	0.018
Myopia SE ≤ -0.5 (n = 315)						
Wearing glasses	0.237 (0.005–0.469)	0.045	0.252 (-0.070–0.574)	0.126	0.319 (0.076–0.562)	0.010
**Model 1**						
Sex	-0.340 (-0.555 to -0.125)	0.002	-0.162 (-0.469–0.145)	0.300	-0.572 (-0.795 to -0.348)	< 0.001
Study time (ref. > 8 hours/day)						
≤ 6 hours/day	-0.903 (-1.352 to -0.455)	<0.001	-0.939 (-1.577 to -0.300)	0.004	-0.591 (-1.056 to -0.126)	0.013
6-8 hours/day	-0.344 (-0.590 to -0.098)	0.006	-0.436 (-0.787 to -0.086)	0.015	-0.296 (-0.552 to -0.041)	0.023
Wearing glasses	0.204 (-0.019–0.426)	0.073	0.227 (-0.090–0.544)	0.160	0.280 (0.049–0.511)	0.018
**Model 2**						
Sex	-0.336 (-0.5552 to -0.121)	0.002	-0.166 (-0.472–0.141)	0.289	-0.567 (-0.791 to -0.344)	< 0.001
Study time (ref. > 8 hours/day)						
≤ 6 hours/day	-0.908 (-1.356 to -0.460)	<0.001	-0.950 (-1.588 to -0.312)	0.003	-0.590 (-1.055 to -0.125)	0.013
6-8 hours/day	-0.352 (-0.599 to -0.106)	0.005	-0.435 (-0.786 to -0.084)	0.015	-0.305 (-0.561 to -0.049)	0.020
Time spent electronic devices for pleasure (ref. > 1 hours/day)						
Not at all	0.152 (-0.229–0.532)	0.434	-0.055 (-0.597–0.487)	0.842	0.158 (-0.237–0.554)	0.432
≤ 1 hours/day	0.123 (-0.115–0.362)	0.311	0.192 (-0.148–0.532)	0.269	0.023 (-0.225–0.271)	0.854
Wearing glasses	0.194 (-0.029–0.417)	0.089	0.212 (-0.105–0.530)	0.190	0.277 (0.046–0.509)	0.019

CI, confidence interval; GPA, grade point average; SE, spherical equivalent

Discussion

This study aimed to determine the prevalence of myopia and low vision among secondary schoolchildren in Vietnam and assessed the effects of wearing glasses on academic performance among children with low vision and myopia. Among the participants, 48.7% and 23.2% had myopia and poor vision, respectively. Although eyeglasses are considered to be a cost-effective way to treat myopia, the empirical evidence of their impact on improving learning outcomes is inconsistent. The results of this study revealed, however, that wearing eyeglasses was significantly associated with improved academic performance among secondary schoolchildren with low vision and myopia in Hue City, Vietnam.

Myopia is considered a significant Asian public health issue, as its prevalence being notably higher in Asia than Western countries [11–13], and is perceived to be a critical health problem in various urbanized areas (e.g., Singapore, Taipei, China, India) [14–17]. The results of this study showed that the prevalence of myopia among the secondary schoolchildren was 48.7%. Similar to our study, refractometry has been performed under non-cycloplegic conditions in other studies, the results of which found that myopia is a common refractive error in schools in Vietnam [11,18]. Another study of rural secondary schoolchildren, however, showed a lower prevalence of students with myopia than what we observed [19]. The prevalence of myopia among Vietnamese schoolchildren is relatively high compared to that in Western countries, but similar to or lower than that in other Asian countries. Prior results from a study in China showed a slightly higher prevalence of myopia among primary and secondary school-aged children [20–23], whereas studies on urban and suburban Indian schoolchildren showed a lower prevalence [24,25]. These differences may be due to differences in subject age, cutoff thresholds for a diagnosis of myopia, or measurement methods (cycloplegic vs. non-cycloplegic). Additionally, the prevalence of low vision (UCVA) in both eyes was 23.3% in our study, slightly higher than that reported in other school-based studies conducted by the same group [26,27]. Ultimately, an accurate comparison of refractive error prevalence rates with other studies is difficult, due to the use of different definitions, instrumentation, and variable use of cycloplegia, as well as differences in other population characteristics, such as ethnicity, sex, and age distribution.

The results of this study also showed that nearly two-thirds (63.2%) of students with myopia and a quarter (25.3%) of students with low vision did not wear glasses. Although multiple studies have emphasized that wearing glasses has a definitive impact on slowing the progression of myopia [28–31], a significant number of students with myopia do not wear glasses. A similarly high and unmet need for refractive correction has been documented in previous studies. A prior study in Vietnam indicated that about two out of three secondary school students with refractive errors have not received the appropriate correction [32]. Among other populations, results from India showed that 75% of children with myopia did not wear glasses [33], while a study from China showed that wearing glasses was observed in only 37% of children with visual impairments. Despite better access to eye doctors and optical shops in urban areas, there are still urban schoolchildren who had untreated refractive errors. A large number of myopic students do not wear glasses in Vietnam. This situation in Vietnam and other low- and middle-income countries could be attributed to reasons that include a lack of comprehensive vision health education, particularly regarding the benefits of glasses in treating refractive errors and insuffficient financal support for vision health promotion. Furthermore, existing evidence indicates that children do not like glasses or are self-conscious of their appearance in them [34]; therefore, having children comply with wearing glasses should be a concern, indicating a need for health education regarding wearing glasses and vision screening, as research has shown that eye health promotion interventions significantly improve eye health knowledge, attitudes and practices of school children [35].

The main finding of this study was the significant effects of wearing glasses on academic performance (e.g., GPA, math and literature scores) among schoolchildren with low vision and myopia. This association remained significant after adjusting for sex, study time, and time spent on electronic devices for pleasure in the low-vision group (model 2). In the myopic group, wearing glasses was significantly associated with the Vietnamese literature score, but not with GPA or math score, after adjusting for sex, study time, and time spent on electronic devices for pleasure. Students with myopia who wore glasses had significantly higher scores in Vietnamese literature than their counterparts. The findings from our study corroborated the those of previous studies in different populations. A study from China revealed that wearing glasses improved the Chinese and math test scores of students with myopia by 0.27 and 0.24 standard deviations, respectively [12], while another study showed that better visual acuity, but not cycloplegia refractive error or wear glasses, was significantly associated with academic performance [36]. This difference might be because academic performance was

measured by standardized examinations that included five subjects, instead of measuring each subject as in our study. Other evidence from a cluster randomized clinical trial in the United States showed that students who wore eyeglasses achieved better reading scores [37]. The association between wearing glasses and academic performance can be explained easily, as being able to see clearly at a distance is crucial for students to learn, especially since most secondary schools often use blackboards or whiteboards as the main tool of instruction, along with digital displays.

The strength of our study lies in the availability of different measures of academic performance, including GPAs, math scores, and literature scores. However, this study had some limitations. First, autorefractometry in the present study was not performed under cycloplegic conditions, and noncycloplegic autorefractometry tends to overestimate the prevalence of myopia in teenagers with active accommodation. Thus, future research should incorporate cycloplegic conditions to improve the accuracy and reliability of myopia assessments. Second, independent variables such as study time and time spent on electronic devices for pleasure were collected through face-to-face interviews, and might have been subject to recall bias. Despite these limitations, this study provides suggestive evidence that improved compliance in wearing glasses may lead to enhanced academic outcomes in secondary schoolchildren. The current findings provide an additional impetus for concerted efforts from government ministries, schools, parents, and healthcare professionals to ensure that no child is denied access to good education because of poor vision.

Acknowledgements

The authors would like to thank Prof. Nguyen Vu Quoc Huy for overarching advice on the research and support of data collection by the teams of the Faculty of Public Health and Department of Ophthalmology of Hue University of Medicine and Pharmacy. We would also like to thank the Department of Education and Training in Thua Thien Hue Province, Vietnam, for granting permission for their schools to participate in this study, the schools and teachers who kindly agreed to administer the questionnaire, and all of the students who generously agreed to participate.

References

1. Pan CW, Ramamurthy D, Saw SM. Worldwide prevalence and risk factors for myopia. Ophthalmic and Physiological Optics. 2012 Jan 1;32(1):3–16.

2. Limburg H, Gilbert C, Hon DN, Dung NC, Hoang TH. Prevalence and Causes of Blindness in Children in Vietnam. Ophthalmology. 2012 Feb 1;119(2):355–61.

3. Morgan IG, Ohno-Matsui K, Saw SM. Myopia. The Lancet. 2012 May 5;379(9827):1739–48.

4. French AN, Ashby RS, Morgan IG, Rose KA. Time outdoors and the prevention of myopia. Exp Eye Res. 2013 Sep 1;114:58–68.

5. Li SM, Li SY, Kang MT, Zhou Y, Liu LR, Li H, et al. Near Work Related Parameters and Myopia in Chinese Children: the Anyang Childhood Eye Study. PLoS One [Internet]. 2015 Aug 5 [cited 2022 Oct 18];10(8):e0134514. Available from: https://journals.plos.org/plosone/article?id=10.1371/journal.pone.0134514

6. Rose KA, Morgan IG, Ip J, Kifley A, Huynh S, Smith W, et al. Outdoor Activity Reduces the Prevalence of Myopia in Children. Ophthalmology. 2008 Aug 1;115(8):1279–85.

7. Xiong S, Sankaridurg P, Naduvilath T, Zang J, Zou H, Zhu J, et al. Time spent in outdoor activities in relation to myopia prevention and control: a meta-analysis and systematic review. Acta Ophthalmol. 2017 Sep 1;95(6):551–66.

8. Ip JM, Saw SM, Rose KA, Morgan IG, Kifley A, Wang JJ, et al. Role of Near Work in Myopia: Findings in a Sample of Australian School Children. Invest Ophthalmol Vis Sci. 2008 Jul 1;49(7):2903–10.

9. Lin Z, Gao TY, Vasudevan B, Ciuffreda KJ, Liang YB, Jhanji V, et al. Near work, outdoor activity, and myopia in children in rural China: the Handan offspring myopia study. BMC Ophthalmol. 2017 Nov 17;17(1).

10. Gilbert C, Foster A. Childhood blindness in the context of VISION 2020--the right to sight. Bull World Health Organ. 2001;79(3):227.

11. Pan CW, Dirani M, Cheng CY, Wong TY, Saw SM. The age-specific prevalence of myopia in Asia: A meta-analysis. Optometry and Vision Science. 2015 Mar 6;92(3):258–66.

12. Huang J, Chen Q. Should myopic students wear eyeglasses?—A regression-discontinuity analysis. https://doi.org/101080/1350485120222094322. 2022;

13. Saw SM, Pan CW, Dirani M, Cheng CY, Wong TY. Is myopia more common in Asians? A systematic review and meta-analysis. Invest Ophthalmol Vis Sci. 2014 Apr 30;55(13):3632–3632.

14. Karuppiah V, Wong L, Tay V, Ge X, Kang LL. School-based programme to address childhood myopia in Singapore. Singapore Med J. 2021 Feb 1;62(2):63–8.

15. Wang Y, Liu L, Lu Z, Qu Y, Ren X, Wang J, et al. Rural-urban differences in prevalence of and risk factors for refractive errors among school children and adolescents aged 6–18 years in Dalian, China. Front Public Health. 2022 Aug 29;10:2910.

16. Tsai DC, Fang SY, Huang N, Hsu CC, Chen SY, Chiu AWH, et al. Myopia Development Among Young Schoolchildren: The Myopia Investigation Study in Taipei. Invest Ophthalmol Vis Sci. 2016 Dec 1;57(15):6852–60.

17. Saxena R, Vashist P, Tandon R, Pandey RM, Bhardawaj A, Gupta V, et al. Incidence and progression of myopia and associated factors in urban school children in Delhi: the North India myopia study (NIM Study). PLoS One. 2017 Dec 1;12(12):e0189774.

18. Wu LJ, You QS, Duan JL, Luo YX, Liu LJ, Li X, et al. Prevalence and Associated Factors of Myopia in High-School Students in Beijing. PLoS One. 2015 Mar 24;10(3):e0120764.

19. Hung HD, Chinh DD, Van Tan P, Duong NV, Anh NQ, Le NH, et al. The Prevalence of Myopia and Factors Associated with It Among Secondary School Children in Rural Vietnam. Clinical Ophthalmology [Internet]. 2020 Apr 22 [cited 2022 Oct 19];14:1079–90. Available from: https://www.dovepress.com/the-prevalence-of-myopia-and-factors-associated-with-it-among-secondar-peer-reviewed-fulltext-article-OPTH

20. Wang SK, Guo Y, Liao C, Chen Y, Su G, Zhang G, et al. Incidence of and Factors Associated With Myopia and High Myopia in Chinese Children, Based on Refraction Without Cycloplegia. JAMA Ophthalmol [Internet]. 2018 [cited 2022 Oct 18];136(9):1017–24. Available from: https://pubmed.ncbi.nlm.nih.gov/29978185/

21. Guo L, Yang J, Mai J, Du X, Guo Y, Li P, et al. Prevalence and associated factors of myopia among primary and middle school-aged students: a school-based study in Guangzhou. Eye 2016 30:6. 2016 Mar 11;30(6):796–804.

22. You QS, Wu LJ, Duan JL, Luo YX, Liu LJ, Li X, et al. Factors Associated with Myopia in School Children in China: The Beijing Childhood Eye Study. PLoS One [Internet]. 2012 Dec 27 [cited 2023 Apr

20];7(12):e52668. Available from: https://journals.plos.org/plosone/article?id=10.1371/journal.pone.0052668

23. Huang Z, Song D, Tian Z, Wang Y, Tian K. Prevalence and associated factors of myopia among adolescents aged 12–15 in Shandong Province, China: a cross-sectional study. Scientific Reports 2024 14:1 [Internet]. 2024 Jul 27 [cited 2024 Aug 5];14(1):1–10. Available from: https://www.nature.com/articles/s41598-024-68076-5

24. Gopalakrishnan A, Hussaindeen JR, Sivaraman V, Swaminathan M, Wong YL, Armitage JA, et al. Prevalence of myopia among urban and suburban school children in Tamil Nadu, South India: findings from the Sankara Nethralaya Tamil Nadu Essilor Myopia (STEM) Study. Ophthalmic and Physiological Optics. 2022 Mar 1;42(2):345–57.

25. Saxena R, Vashist P, Tandon R, Pandey RM, Bhardawaj A, Menon V, et al. Prevalence of Myopia and Its Risk Factors in Urban School Children in Delhi: The North India Myopia Study (NIM Study). PLoS One. 2015 Feb 26;10(2):e0117349.

26. O'Donoghue L, McClelland JF, Logan NS, Rudnicka AR, Owen CG, Saunders KJ. Refractive error and visual impairment in school children in Northern Ireland. British Journal of Ophthalmology. 2010 Sep 1;94(9):1155–9.

27. Paudel P, Ramson P, Naduvilath T, Wilson D, Phuong HT, Ho SM, et al. Prevalence of vision impairment and refractive error in school children in Ba Ria - Vung Tau province, Vietnam. Clin Exp Ophthalmol. 2014 Apr 1;42(3):217–26.

28. Lam CSY, Tang WC, Zhang HY, Lee PH, Tse DYY, Qi H, et al. Long-term myopia control effect and safety in children wearing DIMS spectacle lenses for 6 years. Scientific Reports 2023 13:1 [Internet]. 2023 Apr 4 [cited 2023 Aug 8];13(1):1–10. Available from: https://www.nature.com/articles/s41598-023-32700-7

29. Walline JJ, Lindsley K, Vedula SS, Cotter SA, Mutti DO, Twelker JD. Interventions to slow progression of myopia in children. Cochrane Database of Systematic Reviews [Internet]. 2011 Dec 7 [cited 2023 Aug 8];(12). Available from: https://www.cochranelibrary.com/cdsr/doi/10.1002/14651858.CD004916.pub3/full

30. Bao J, Yang A, Huang Y, Li X, Pan Y, Ding C, et al. One-year myopia control efficacy of spectacle lenses with aspherical lenslets. British Journal of Ophthalmology [Internet]. 2022 Aug 1 [cited 2023 Aug 8];106(8):1171–6. Available from: https://bjo.bmj.com/content/106/8/1171

31. Ma Y, Congdon N, Shi Y, Hogg R, Medina A, Boswell M, et al. Effect of a Local Vision Care Center on Eyeglasses Use and School Performance in Rural China: A Cluster Randomized Clinical Trial. JAMA Ophthalmol [Internet]. 2018 Jul 1 [cited 2022 Oct 25];136(7):731–7. Available from: https://jamanetwork.com/journals/jamaophthalmology/fullarticle/2680115

32. Anh VT, Fabrizio D, Xuan LTT, Van PT Van, Nga TTH. Survey of Refractive Errors in Secondary School and Refractive Errors Based- School Screening in Vietnam. Open Access Maced J Med Sci [Internet]. 2022 Sep 30 [cited 2024 Aug 26];10(E):1911-1918. Available from: https://oamjms.eu/index.php/mjms/article/view/10273

33. Saxena R, Gupta V, Prasad P, Bhardwaj A, Vashist P. Prevalence of myopia and its risk factors in rural school children in North India: the North India myopia rural study (NIM-R Study). Eye 2021 36:10. 2021 Oct 13;36(10):2000–5.

34. Gogate P, Mukhopadhyaya D, Mahadik A, Naduvilath T, Sane S, Shinde A, et al. Spectacle compliance amongst rural secondary school children in Pune district, India. Indian J Ophthalmol. 2013 Jan;61(1):8.

35. Paudel P, Yen PT, Kovai V, Naduvilath T, Ho SM, Giap NV, et al. Effect of school eye health promotion on children’s eye health literacy in Vietnam. Health Promot Int [Internet]. 2019 Feb 1 [cited 2024 Aug 26];34(1):113–22. Available from: https://dx.doi.org/10.1093/heapro/dax065

36. Jan C, Li SM, Kang MT, Liu L, Li H, Jin L, et al. Association of visual acuity with educational outcomes: a prospective cohort study. British Journal of Ophthalmology. 2019 Nov 1;103(11):1666–71.

37. Neitzel AJ, Wolf B, Guo X, Shakarchi AF, Madden NA, Repka MX, et al. Effect of a Randomized Interventional School-Based Vision Program on Academic Performance of Students in Grades 3 to 7: A Cluster Randomized Clinical Trial. JAMA Ophthalmol. 2021;139(10).
