# Supplementary material for: Impact of spectacle use on academic performance among Vietnamese adolescents with reduced visual acuity and myopia: A school-based study
Source: PLoS One. 2025 May 12;20(5):e0322534. doi: 10.1371/journal.pone.0322534 (PMC12068628; doi:10.1371/journal.pone.0322534)
Supplement: Attachment — Submitted filename: ResponseToReviewers_PONE-D-24-39987R2_0217.docx [file pone.0322534.s005.docx]

**PLOS ONE Decision: Revision required [PONE-D-24-39987R1] - [EMID:e61f0d93096e664e]**

Reviewer #1: The authors have provided thorough and thoughtful responses to the reviewers' comments. Sentence amendment and word replacement were updated, and the explanation for retaining "sex" is concise and justified. Additional analysis on study time was performed and separate tables were made to clarify the sample grouping. The explanations regarding "wearing glasses" as an independent variable also address the reviewer’s methodological concerns. The authors have commendably acknowledged the limitations of the study's cross-sectional nature while performing an additional analysis to investigate performance differences among groups. The explanation regarding future research is reasonable but could be expanded to suggest specific longitudinal or interventional study designs that would help address this concern.
Below are detailed evaluations and suggestions for further refinement:

Major Concerns:

1. Separate tables were made to clarify the sample grouping. But there is a lack of exploration into the possibility of differences in outcomes between the VA group and the myopia group.

Response:

Thank you for expressing this concern. The distributions of VA group and myopia group differ, as shown in Table 3. We have revised the sentence in the Discussion section clearly stating that the associations between spectacle use and academic achievements were stronger among the students with reduced VA (Table 4) than among students with myopia (Table 5).” (Page 11, Lines 217-219)

2. For the 35% of the myopia group without reduced visual acuity (VA), the importance of wearing glasses needs to be demonstrated.

Response:

In response to the reviewer’s comments, there are several reasons why students with myopia may not experience noticeable visual impairment. One factor is the accommodative mechanism and the use of non-cycloplegic measurements, which we have already acknowledged in the limitations section (Page 11, Lines 234-238). Furthermore, we have tested the association between wearing spectacles and academic performance among students with myopia but no reduced VA (n=174). Results showed that wearing spectacles was significantly associated with improved academic performance among students with myopia but reduced VA, with increased math scores (β = 1.108; *P* = 0.023) compared with those of students not wearing spectacles, remained significantly associated after adjusting for sex, study time, and time spent on electronic devices for pleasure. Thus, use of spectacle is beneficial to students with myopia even without reduced VA.

Minor Concerns:

1. In Page 3, Lines 65-66, the authors updated the global data that “only 36% of people with distance vision impairment due to refractive errors have access to appropriate spectacles” The citation needs to be added.

Response:

In response to the reviewer’s comments, the sentence was cited (ref. 13, in Page 3, Lines 64-65)
